# Assessment of changes in quality of life after thyroid surgery in older adults. A prospective study

**DOI:** 10.3389/fendo.2026.1967551

**Published:** 2026-10-14

**Authors:** Vittorio Falco, Yanina Morosán Allo, Carina Parisi, Cristina Faingold, Gabriel Fideleff, Maximiliano Lo Tartaro, Tomas Meroño, Torquil Watt, Victor Brun Boesen, Ulla Feldt-Rassmussen, Gabriela Brenta

**Affiliations:** 1 Department of Medical Endocrinology and Metabolism, Unidad Asistencial Dr. César Milstein, Buenos Aires, Argentina; 2 Department of Biochemistry, Unidad Asistencial Dr. César Milstein, Buenos Aires, Argentina; 3 Department of Surgery, Unidad Asistencial Dr. César Milstein, Buenos Aires, Argentina; 4 Biomarkers and Nutrimetabolomics Laboratory, Department of Nutrition, Food Sciences and Gastronomy, Nutrition and Food Safety Research Institute (INSA-UB), Faculty of Pharmacy and Food Sciences, University of Barcelona, Barcelona, Spain; 5 Medical and Translational Science, Novo Nordisk, Søborg, Denmark; 6 Department of Medicine, Zealand University Hospital, Køge, Denmark; 7 Department of Growth and Reproduction, Rigshospitalet, Copenhagen University Hospital, Copenhagen, Denmark; 8 Institute of Clinical Medicine, Faculty of Health and Clinical Sciences, Copenhagen University, Copenhagen, Denmark

**Keywords:** older adults, quality of life, ThyPRO, thyroid nodular disease, thyroid surgery

## Abstract

**Objective:**

Referral of older adults with thyroid nodular disease to surgery remains a challenge. We measured the impact of thyroid surgery on thyroid-related quality of life among older adults with nodular thyroid disease to aid decision-making.

**Methods:**

In this prospective study, all patients aged> 60 years undergoing thyroid surgery for nodular thyroid disease completed the short-form Thyroid-Related Quality of Life (QoL) Patient-Reported Outcome (ThyPRO-39), expanded to include the full 85-item ThyPRO scales for physical thyroid symptoms, at admission and 6–12 months after surgery. Thyroid function testing, histological reports, and surgery extension were recorded. Wilcoxon tests were corrected for multiple comparisons, and linear mixed models were employed in the statistical analyses.

**Results:**

Of 88 patients (81% female, age 71 ± 6 years), 31 had malignant and 57 benign nodules. After surgery, patients showed a significant improvement (p < 0.01) in 10 of the 13 ThyPRO scales, four physical symptoms, two mental well-being scales (Anxiety and emotional susceptibility), all QoL Impairment scales, and the ThyPRO-39 Composite scale (30 ± 17 vs. 21 ± 13, p < 0.01). Improvements in physical symptoms and QoL impairment scales were observed primarily in the benign nodules group (*p* for interaction < 0.05). Although both lobectomy and total thyroidectomy improved QoL, lobectomized patients maintained T3 concentrations comparable to baseline.

**Conclusion:**

Surgery improved most of the ThyPRO domains in older adults with nodular disease. Furthermore, goiter-related symptoms that impaired daily life and overall QoL improved primarily in benign disease. ThyPRO scores improved after both hemithyroidectomy and total thyroidectomy.

## 1 Introduction

Thyroid nodular disease is more frequent in older adults than in the general population (1) and is characterized by benign nodules that may cause local symptoms of neck compression (2) or by malignant nodules that are advanced at initial presentation (3). The paradox in older adults is that they have a higher frequency of thyroid nodules than younger adults, but the likelihood that a thyroid nodule harbours malignancy is much lower (1). Therefore, referrals of older patients for thyroid surgery should be made carefully, prioritizing those with nodular goiter that develop compressive symptoms and those with potentially aggressive malignant nodules.

In older adults undergoing thyroid surgery, deterioration in quality of life may be caused by multiple factors: the need for hormone replacement therapy, post-surgical complications, adjuvant treatment with radioactive iodine (RAI), and persistent fear of disease recurrence. These factors negatively affect psychological well-being and social functioning (4). A less invasive surgical option, such as hemithyroidectomy or active surveillance (5) may be preferred in the presence of comorbidities (6, 7) or a low risk of recurrence in cases of malignant thyroid disease (8).

On the other hand, thyroid surgery may improve quality of life, particularly in older patients with long-standing benign goiter and compressive symptoms, or even in patients with nodules with proven malignant cytology who may be ideal candidates for active surveillance but still have concerns about cancer.

Quality of life (QoL), understood as a subjective, dynamic, and multidimensional experience, encompasses physical, psychological, and social well-being. Generic QoL questionnaires, such as the Short Form Health Survey (SF-36) or the EuroQoL EQ-5D, have yielded inconsistent results for assessing QoL after thyroid surgery (9). However, a disease-specific questionnaire, such as the ThyPRO (Thyroid Patient-Reported Outcome) developed by Watt et al. (10, 11) for assessing QoL in patients with benign thyroid disease, has been used to evaluate QoL in both benign and malignant diseases (12, 13). Furthermore, the last American Thyroid Association (ATA) guidelines on thyroid cancer emphasized individualized assessment of the risks and benefits of each intervention and the integration of QoL and patient-reported outcomes (PROs) into the decision-making framework (5).

Since the indication of surgical treatment in older adults with thyroid disease continues to represent a clinical challenge, the effect of surgery on QoL should be taken into account in the decision-making in this age group.

In this study, we aimed to evaluate the impact of thyroid surgery on thyroid-related QoL in older adults with nodular thyroid disease. Additionally, we aimed to assess the effects of surgical extension on QoL in relation to the patient’s peripheral thyroid hormone levels.

## 2 Materials and methods

### 2.1 Study design

This prospective, longitudinal study evaluated all consecutive patients aged 60 years or older referred for thyroid surgery at a university-affiliated tertiary-care teaching hospital in Buenos Aires from June 2018 to 2024. After obtaining informed consent, healthcare personnel administered the ThyPRO questionnaire on the day of surgical admission, and patients completed it under supervision. We collected demographic data, comorbidities, history of thyroid pathology, and other relevant medical conditions. In addition, we obtained blood samples to assess thyroid function and calcium and phosphate homeostasis.

Surgical indication in patients with cytologically benign tumors was based on tumor size and compressive symptoms. For the rest of the population, the decision was made based on an indeterminate, suspicious-for-malignancy, or malignant cytological report. Patients referred for other reasons, such as hyperthyroidism and hyperparathyroidism with concomitant thyroid nodules, were also included. We excluded patients who could not complete the questionnaire at baseline or during follow-up. The type of surgery, either hemithyroidectomy or total thyroidectomy, was decided before surgery by an interdisciplinary medical team, taking into consideration the presence of one or more nodules, the size and extent of the lesion, age, and comorbidities for each patient. The same two surgeons treated all these patients.

After surgery, data on the type of surgery performed, hemithyroidectomy or total thyroidectomy, surgical complications, and histological findings, benign or malignant tumor, were recorded.

The questionnaire was completed again at an outpatient consultation or by phone among patients who returned home to a distant location, 6 to 12 months after surgery. Although we initially planned assessment at approximately 6 months, some participants required repeated contact attempts because they were no longer receiving routine follow-up at the institution, resulting in later assessments within the predefined follow-up window.

We collected laboratory results for thyroid function and calcium and phosphate metabolism, obtained over the 4 weeks preceding the consultation.

The questionnaire results, patient history, physical examination, surgical and pathological findings, and biochemical results were uploaded to an electronic database, with data integrity controls.

### 2.2 Quality of life assessment

The Spanish version of the short-form Thyroid-Related Quality of Life (QoL) Patient-Reported Outcome (ThyPRO-39) (14), expanded to include the full 85-item ThyPRO scales for physical thyroid symptoms, was used. Throughout this manuscript, this instrument is referred to as “ThyPRO.”

The ThyPRO questionnaire covers thyroid-related physical and mental symptoms, the impact of thyroid disease on participation in social and daily activities, and overall QoL. The instrument consists of four full ThyPRO-85 scales covering physical symptoms (Goiter-, Hyperthyroid-, Hypothyroid-, and Eye symptoms), five ThyPRO-39 scales covering tiredness and mental health (Tiredness, Cognition, Depressiveness, Anxiety, and Emotional susceptibility), three scales covering areas of life impacted by thyroid disease (Impaired social life, Impaired daily life, and Cosmetic complaints), and a single item assessing Overall QoL-impact. A composite score is based on 22 items from the Tiredness, Cognition, Anxiety, Depressiveness, Emotional Susceptibility, Impaired Social life, Impaired Daily Life and Overall QoL-impact.

Each item is rated on a 5-point Likert scale from 0 (not at all) to 4 (a lot), referring to the last 4 weeks. Each scale is scored as a summary score (with inversion of positively formulated items) and linearly transformed to a range in which higher scores indicate poorer health status.

In brief, ThyPRO scores range from 0 to 100, with higher scores indicating poorer QoL.

### 2.3 Preoperative biochemical measurements

Venous blood samples were obtained in the early morning after an overnight fast, prior to surgery. Serum samples were processed within one hour of collection for calcium and albumin measurements. A second serum sample was centrifuged, aliquoted, and stored at −20 °C until analysis, which was performed within 7 days. This sample was used to determine serum concentrations of thyrotropin (TSH), free thyroxine (free T4), total triiodothyronine (T3), intact parathyroid hormone (PTH), and 25-hydroxyvitamin D [25(OH)D].

All hormone measurements were performed using an automated chemiluminescent immunoassay system (ADVIA Centaur XPT, Siemens Healthineers, Germany). The reference intervals were 0.3–6.0 mIU/L for TSH, 7.7–23.3 pmol/L (0.6–1.8 ng/dL) for free T4, 1.23–2.93 nmol/L (80–190 ng/dL) for total T3, and 1.3–8.1 pmol/L (12.4–76.8 pg/mL) for PTH. Inter-assay coefficients of variation (CV) ranged from 3.0% to 7.1% for TSH, 4.0% to 8.0% for free T4, 5.0% to 9.0% for T3, 3.8% to 6.2% for PTH, and 6.6% for 25(OH)D.

Vitamin D status was defined as deficiency (<50 nmol/L [20 ng/mL]), insufficiency (50–75 nmol/L [20–30 ng/mL]), or sufficiency (>75 nmol/L [30 ng/mL]).

Serum calcium and albumin were measured using an automated colorimetric method (Cobas C311 Chemistry Analyzer, Roche Diagnostics, Switzerland). Serum calcium levels were corrected for albumin concentration using the following formulas: corrected calcium (mmol/L) = serum calcium + [0.02 × (40 − albumin [g/L])], corrected calcium (mg/dL) = serum calcium + [0.8 × (4 − albumin [g/dL])].

Hypocalcemia was defined as a serum calcium level ≤8.5 mg/dL (≤2.12 mmol/L), and severe hypocalcemia as ≤7.5 mg/dL (≤1.87 mmol/L). Postsurgical hypoparathyroidism (hPTpost) was defined as a PTH level ≤ 1.3 pmol/L (12.1 pg/mL) (according to the laboratory reference range).

### 2.4 Postoperative biochemical assessment

We obtained postoperative biochemical tests during routine clinical follow-up. Samples were analyzed either at the study institution or at external laboratories selected by the participants. Consequently, we did not use a uniform analytical platform, and we could not ascertain the specific assay methodology for all measurements. We interpreted each result using the reference range provided by the laboratory that performed the analysis.

### 2.5 Ethical considerations

The institutional review committee approved the study, and all participants provided written informed consent.

### 2.6 Statistical analysis

Variables were classified as continuous or categorical based on data distribution.

The Wilcoxon Signed-rank test was used to compare ThyPRO Scores at Baseline and at follow-up 6–12 months after treatment, and the interaction term was modeled in linear mixed models adjusted for time and histology type (Benign vs Malignant), with subject-specific random effects.

The comparison of baseline and follow-up thyroid function test results according to surgical extension was performed using the Wilcoxon test.

All *p<0.05* was considered significant.

Statistical analyses were performed using SPSS 17.0 (IBM, Chicago, Ill., USA).

## 3 Results

### 3.1 Characteristics of the study population

Of 88 patients (81% female, age 71 ± 6 years, median 70 (IQR, 66–75 years) included in this study (**Figure 1**), 34.1% were referred for surgery for compressive symptoms, 24% for indeterminate cytology, 22% for malignant cytologic findings, and the rest for nodule size, unsatisfactory cytology, hyperthyroidism, or hyperparathyroidism. Most patients underwent a total thyroidectomy (70.5%), while 27% were referred to a hemithyroidectomy. Two patients were referred for lymph node resection and a subtotal thyroidectomy. After surgery, 31 patients were identified with malignant and 57 with benign thyroid nodules.

**Figure 1:**
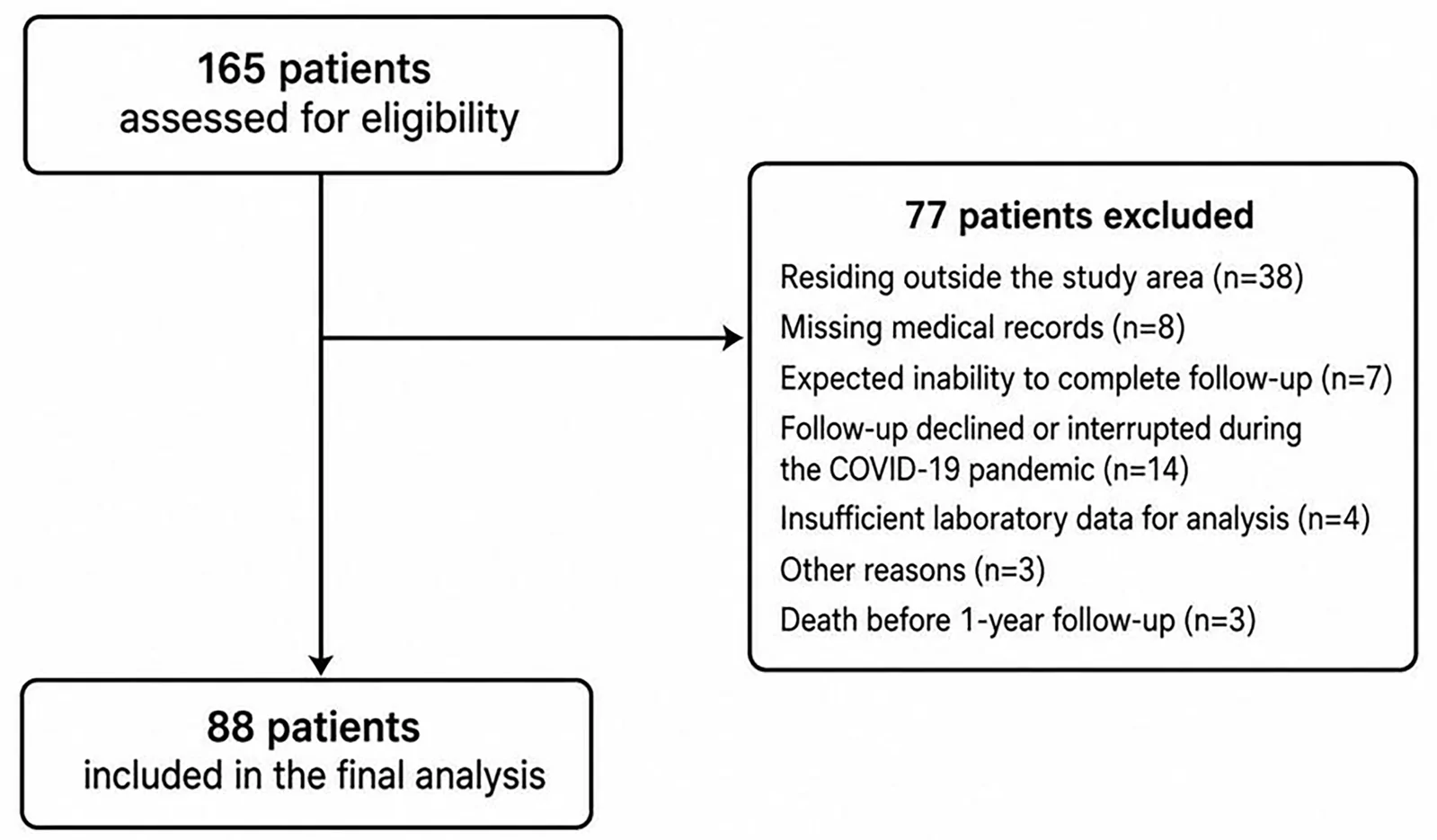
Flowchart of patient selection.

Patients’ characteristics, surgical indication, surgical extension, histology, and perioperative morbidity are shown in **Table 1**.

**Table 1:** Patients’ characteristics, surgical indication (SI), surgical extension, histology, and perioperative morbidity (n: 88).

Characteristic	Value (n = 88)
Age, y mean ± SD	71 ± 6
Male, n (%) Female, n (%)	17 (19) 71 (81)
Smoking, n (%)	13/87 (15)
Diabetes, n (%)	17 (19)
Hypothyroidism, n (%)	24 (27)
BMI, kg/m^2^, mean ± SD	30 ± 5.6
SI: Compressive symptoms, n (%)	30 (34.1)
SI: Nodule size, n (%)	8 (9.1)
SI: Unsatisfactory cytology, n (%)	6 (6.8)
SI: Indeterminate cytology, n (%)	21 (24)
SI: Malignant cytology, n (%)	19 (22)
SI: Hyperthyroidism, n (%)	1 (1)
SI: Hyperparathyroidism, n (%)	3 (3)
Benign histology, n (%)	57 (65)
Malignant histology, n (%)	31 (35)
Hemithyroidectomy, n (%)	24 (27)
Total Thyroidectomy, n (%)	62 (70.5)
Hypoparathyroidism, n (%)	1 (2)

Among patients whose nodules were benign on histology, the surgical indications were: 49% for compressive symptoms, 14% for nodule size, 7% for non-diagnostic cytology, 15% for indeterminate cytology, 8% for malignant cytology, 1% for hyperthyroidism, and 5% for hyperparathyroidism. In patients with thyroid cancer on histology, the surgical indications were: 9% for compressive symptoms, 6% for non-diagnostic cytology, 38% for indeterminate cytology, and 45% for malignant cytology. No major surgical complications such as permanent hypoparathyroidism or irreversible laryngeal nerve damage were reported at the follow-up in this cohort.

### 3.2 Assessment of QoL before and after surgery

At 6–12 months, median ThyPRO scores were lower than baseline across most domains, indicating an improvement (**Table 2**, **Figure 2**).

**Table 2:** ThyPRO scores at baseline and at follow-up 6–12 months after treatment.

Domains	Baseline (median Q1-Q3)	6–12 months (median Q1-Q3)	Adjusted *p* value	BvsM interaction
Goitre	18 (7-38)	2 (0-7)	**0.000**	**0.013**
Hyperthyroidism Symptoms	19 (6-36)	6 (0-12.5)	**0.000**	**0.025**
Hypothyroidism Symptoms	25 (6-42)	12.5 (0-25)	**0.000**	**0.036**
Eye Symptoms	12.5 (4-25)	0 (0-6)	**0.000**	**0.005**
Tiredness	46 (25-58)	33 (17-58)	0.084	0.971
Cognition	14 (7-37)	7 (1-29)	0.066	0.168
Anxiety	34 (10-56)	10 (1-26)	**0.000**	0.421
Depressiveness	29 (14-45)	29 (14-35)	0.051	0.366
Emotional Susceptibility	36 (21-58)	28 (13-36)	**0.001**	0.351
Impaired Social Life	0 (0-17)	0	**0.003**	0.899
Impaired Daily Life	0 (0-15)	0 (0-7)	**0.009**	0.287
Cosmetic Complaints	1 (1-21)	1(1-1)	**0.00**	0.624
Overall quality of life impact	12.5 (0-50)	0	**0.001**	**0.006**
Composite QoL score	27 (18-39)	17 (12.5-26)	**0.000**	0.173

B, benign thyroid nodular disease; M, malignant thyroid nodular disease.

ThyPRO: The Spanish version of the short-form Thyroid-Related Quality of Life (QoL) Patient-Reported Outcome (ThyPRO-39), expanded to include the full 85-item ThyPRO scales for physical thyroid symptoms.

Bold numbers denote significant *p* values.

**Figure 2:**
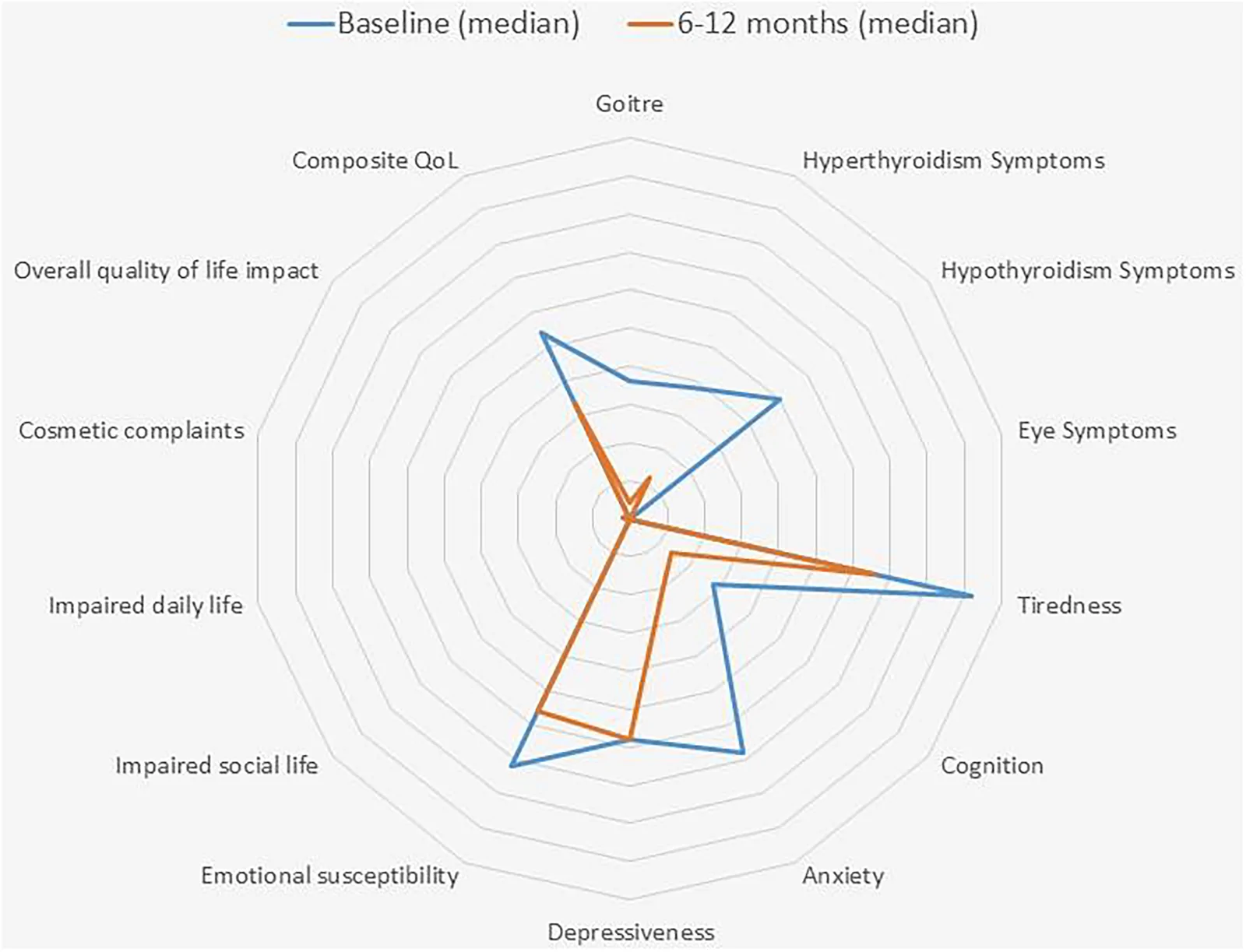
Improvement in ThyPRO scores after surgery in patients with nodular thyroid disease. The blue line depicts the baseline scores, while the red one depicts the postsurgical scores after 6–12 months. ThyPRO: The Spanish version of the short-form Thyroid-Related Quality of Life (QoL) Patient-Reported Outcome (ThyPRO-39), expanded to include the full 85-item ThyPRO scales for physical thyroid symptoms.

Goitre Symptoms decreased from 18 (Q1–Q3: 7–38) to 2 (0–7), Hyperthyroid Symptoms from 19 (6–36) to 6 (0–12.5), Hypothyroid Symptoms from 25 (6–42) to 12.5 (0–25), and Eye Symptoms from 12.5 (4–25) to 0 (0–6) (all adjusted *p* < 0.001), with significant baseline-by-month interaction effects.

Anxiety and Emotional Susceptibility scores were also reduced, with anxiety declining from 34 (10–56) to 10 (1–26) (adjusted *p* < 0.001) and emotional susceptibility from 36 (21–58) to 28 (13–36) (adjusted *p* = 0.001).

Overall QoL Impact decreased from 12.5 (0–50) at baseline to 0 at follow-up (adjusted *p* = 0.001), accompanied by a significant interaction effect, while the Composite QoL Score decreased from 27 (18–39) to 17 (12.5–26) (adjusted *p* < 0.001).

In contrast, changes in Tiredness, Cognitive Complaints, and Depressiveness were not statistically significant.

The Impaired Social Life and Impaired Daily Life domains showed statistically significant adjusted *p*-values, despite median scores remaining at zero.

In summary, after surgery, patients showed significant improvements (*p* < 0.01) in four physical symptoms, two mental well-being scales (Anxiety and emotional susceptibility), all QoL Impairment scales, and the ThyPRO-39 Composite scale.

Improvements in physical symptoms and QoL impairment scales were observed mainly in the benign group (interaction *p* < 0.05) (**Table 2**, **Figure 1**).

No statistically significant differences in score improvement were found between patients undergoing hemithyroidectomy (n=24) and those undergoing total thyroidectomy (n=62); all interaction *p*-values were >0.05.

### 3.3 Thyroid function tests according to the surgical extension

Biochemical assessment at baseline and follow-up, stratified by surgical extension, in 69 patients with available thyroid function testing at 6–12 months, revealed that free T4 concentrations increased, whereas total T3 concentrations decreased significantly only in patients who underwent total thyroidectomy. In contrast, total T3 concentrations were preserved in patients who underwent hemithyroidectomy (**Table 3**).

**Table 3:** Baseline and follow-up (Post) Thyroid function tests, PTH, and Vitamin D concentrations in the whole population and according to surgical extension.

	Baseline whole population	Post whole population	Baseline total thyroidectomy	Post total thyroidectomy	Baseline hemi-thyroidectomy	Post hemi-thyroidectomy
TSH mIU/L	2.15 ± 1.8	3.66 ± 8.4	1.96 ± 1.6	3.99 ± 9.7	2.72 ± 2.5	2.69 ± 1.6
FT4 pmol/L (ng/dL)	14.4 ± 1.3 (1.12 ± 0.1)	15.8 ± 2.6 (1.23 ± 0.2) *	14.5 ± 1.8 (1.13 ± 0.1)	14.0 ± 1.3 (1.09 ± 0.1)	14.0 ± 1.3 (1.09 ± 0.1)	15.7 ± 1.3 (1.22 ± 0.1)
Total T3 nmol/L (ng/dL)	1.57 ± 0.55 (102 ± 36)	1.17 ± 0.54 (76.1 ± 35) *	1.66 ± 0.55 (108 ± 36) ^&^	1.16 ± 0.52 (75.5 ± 34)	1.32 ± 0.46 (86 ± 30)	1.23 ± 0.59 (79.7 ± 38)
Calcaemia mmol/L (mg/dL)	2.30 ± 0.13 (9.2 ± 0.5)	2.30 ± 0.15 (9.2 ± 0.6)	2.33 ± 0.13 (9.3 ± 0.5)	2.30 ± 0.18 (9.2 ± 0.7)	2.30 ± 0.15 (9.2 ± 0.6)	2.35 ± 0.10 (9.4 ± 0.4)
PTH pmol/L (pg/mL)	4.56 ± 2.65 (43 ± 25)	4.31 ± 2.46 (40.7 ± 23.2)	4.59 ± 2.44 (43.3 ± 23)	4.48 ± 2.54 (42.3 ± 24)	4.77 ± 3.07 (45 ± 29)	3.87 ± 1.59 (36.5 ± 15)
VitD nmol/L (ng/mL)	74.5 ± 25.0 (29.8 ± 10)	88.8 ± 27.5* (35.5 ± 11)	76.0 ± 27.5^&^ (30.4 ± 11)	90.5 ± 27.5 (36.2 ± 11)	68.0 ± 20.0 (27.2 ± 8)	84.8 ± 25 (33.9 ± 10)

Results are expressed as mean ± standard deviation (SD).

*p<0.01 between pre vs post whole population.

^&^ p<0.01 between pre vs post total thyroidectomy.

SI units are presented as primary units, with conventional units in brackets.

Among 16 patients with hemithyroidectomy, 9 (56%) were on replacement therapy, whereas all 53 patients with total thyroidectomy received it.

## 4 Discussion

In this study, older patients with both malignant and benign nodular goitre showed improved thyroid-related QoL after thyroid surgery on 10 of the 13 ThyPRO scales, including overall QoL impact. In patients with benign nodules, however, improvements in the physical symptoms and QoL impairment scales were more pronounced than in those patients with thyroid cancer. A similar improvement in QoL after surgery was observed among patients referred for total thyroidectomy or hemithyroidectomy. Furthermore, patients who underwent hemithyroidectomy maintained serum T3 levels, whereas those who underwent total thyroidectomy did not.

As noted, patients with both benign and malignant histology showed improvements in health-related QoL scores, although patients with benign histology had a greater benefit. Because the main surgical indication in this group was compressive symptoms, it is understandable that QoL improved after surgery, as those symptoms resolved. This objective improvement in the ThyPRO score may provide reassuring guidance when considering submission of an older patient for an invasive procedure. A meta-analysis (15) of 496 patients with benign non-toxic goitre, with a mean age of 49 years (SD ± 14.5), evaluated surgical-related outcomes using ThyPRO. The main findings were that QoL improved across all 13 ThyPRO domains at 6 months post-thyroid surgery compared with baseline, particularly in overall QoL, goitre symptoms, and tiredness. Several other studies evaluated patients with an average age of 43.30 ± 11.6 years (18) and 49.8 years (19), and reported that thyroid surgery improves QoL in patients with benign goitre. In some of these studies, younger age was associated with larger improvement of QoL after surgery (16, 17). Furthermore, in the study by Azaria et al. (17), a significant improvement across nearly all domains of the ThyPRO-39 questionnaire was observed after thyroidectomy in a cohort of patients with benign goitre, with no postoperative complications adversely affecting QoL. These findings align with our study, although the main difference from previous studies is that we included only patients aged 60 years and older. In this age group, defining eligibility for surgery is particularly challenging, as increased frailty and concomitant comorbidities may outweigh the benefits of an invasive intervention (18).

However, the improvement observed in patients with malignant histology should be interpreted differently. The indication for thyroid surgery in older adults can also be difficult to determine in patients with a suspicious or malignant nodule. Some patients may experience a decline in QoL related to diagnostic confirmation and fear of recurrence during follow-up, while others may perceive an improvement associated with relief of disease burden after surgical resection. These divergent responses could contribute to the variability observed in QoL assessments among patients with malignant histology. Given the heterogeneity of published strategies for assessing QoL during follow-up of patients with thyroid cancer, a recent systematic review identified younger age as the factor most consistently associated with a higher fear of cancer recurrence, both in patients with differentiated thyroid cancer and in cancer survivors in general (19). In a study of older adults (n: 2007) with a thyroid cancer diagnosis, specific thyroid cancer deaths were 3.8%, while 12.9% died of other causes. It was concluded that patients at high risk for mortality from competing causes should be promptly identified before planning a diagnostic workup of a thyroid nodule in an older patient (20). It is also worth noting that thyroid diseases are highly prevalent among older adults, and with increasing life expectancy, the number of thyroidectomies performed in this population is likely to rise (21). In this context, once the risk of complex surgery is outweighed, eligible patients in good health should not be denied surgery. Thyroid surgery in selected patients aged 75 years and older was a safe procedure in a study that compared 2030 patients, stratified by age into young, older, and elderly groups. The risk of reintervention for bleeding was higher, but there were no differences in terms of hypocalcaemia or recurrent laryngeal nerve palsy (22). In another retrospective cohort of 313 patients aged 75 years or older, length of stay was longer than in matched controls, but the complication rate was similar. In the current study, we observed no clinically relevant complications, allowing ThyPRO responses to be assessed without interference. Taken together, the clear demonstration of improved QoL after surgery in the present study, along with the findings of the studies described above, may support referral for thyroid surgery in patients with thyroid cancer who are eligible for surgery despite advanced age.

As mentioned, an improvement in postoperative quality of life in patients with symptomatic goitre and/or thyroid cancer was observed in the present study independently of the surgical strategy. However, there is still a debate about the extent of the procedure, particularly whether less invasive surgery can offer benefits comparable to those of a total thyroidectomy. In this context, hemithyroidectomy has emerged as a promising alternative (23). A retrospective study in middle-aged patients with thyroid cancer predominance, comparing the two procedures using ThyPRO questionnaire at least 6 months following surgery, concluded that patients who underwent lobectomy reported better overall QoL on self-assessment than those who underwent total thyroidectomy, and that the need for thyroid hormone replacement was the main factor associated with worse QoL (24) No differences in results were observed, however, between patients with benign and malignant histology. By contrast, in the present study, older patients who underwent lobectomy had similar ThyPRO scores to patients who underwent total thyroidectomy, and benign goiter was associated with better QoL outcomes than thyroid cancer. These discrepancies may reflect differences in the populations and study designs. In this study, we used a prospective approach with a linear mixed-model analysis that flexibly handles correlated and unbalanced data and accounts for individual- or group-level variability through random effects. Furthermore, the comparable PRO scores observed in our study suggest that selecting hemithyroidectomy in patients with compressive nodular goiter who have traditionally been referred for total thyroidectomy, even at advanced age, may be as effective at relieving compressive symptoms (5).

Regarding peripheral thyroid hormone levels, T3 values were preserved in the cohort of patients with hemithyroidectomy. Although T3 levels were not associated with ThyPRO (data not shown), lower T3 levels in thyroidectomized patients can lead to symptoms and complaints (8). In patients with hypothyroidism after total thyroidectomy, combination therapy with levothyroxine and triiodothyronine is often required; however, difficulties in pharmacologic management (25) and uncertain benefits on ThyPRO scores (26) limit its clinical advantage. Therefore, maintaining residual thyroid function is essential to preserve postoperative serum T3 levels, as indicated by studies comparing lobectomy and total thyroidectomy (27).

Several limitations warrant consideration. First, the relatively small sample size may have limited the ability to detect significant differences between the hemithyroidectomy and total thyroidectomy groups. Nevertheless, an important strength of the study is its prospective design, in which each patient served as their own control, enabling a linear mixed-effects model to evaluate associations over time. Compared with simple paired analyses, such as the Wilcoxon signed-rank test, linear mixed-effects models are conditional (subject-specific) tests that allow the inclusion of interaction terms.

Another limitation is the broad follow-up window and the lack of exact assessment timing for the QoL assessment. Furthermore, fewer patients completed thyroid function testing at the end of follow-up than completed the ThyPRO questionnaire. This discrepancy was largely attributable to geographic barriers, as some patients returned to their home provinces in Argentina and could participate in telephone interviews but could not undergo concurrent laboratory testing. We acknowledge the lack of a standardized laboratory methodology for postoperative biochemical measurements, as some tests were performed at external laboratories and analytical platform information was unavailable. Nevertheless, we interpreted results according to the assay-specific reference ranges provided by each laboratory.

In addition, the study did not include a formal geriatric or comorbidity assessment score to objectively quantify baseline surgical risk. However, all patients underwent routine preoperative clinical evaluation in accordance with institutional standards, and the team carefully assessed surgical indications to avoid unnecessary procedures. Another limitation is the lack of age-category stratification. However, the median age at surgery was 70 years (IQR, 66–75 years), indicating that 50% of participants were between 66 and 75 years, which may support extrapolation to similar age groups and inform clinical decision-making for patients uncertain about surgery.

Finally, surgical complications were minimal in this cohort; therefore, the generalizability of these outcomes may depend on complication rates and the surgical expertise of individual institutions, which should be considered when deciding whether to perform thyroid surgery locally or at specialized referral centers.

## 5 Conclusions

In older adults with nodular disease, surgery improved most of the problems related to physical thyroid symptoms, mental well-being and function, QoL impairment, and overall QoL impact.

ThyPRO scores improved after both hemithyroidectomy and total thyroidectomy, with no evidence of a differential preoperative-to-postoperative change between procedures.

## Data Availability

The raw data supporting the conclusions of this article will be made available by the authors, without undue reservation.
